# Statin initiation and treatment non-adherence following a first acute myocardial infarction in patients with inflammatory rheumatic disease versus the general population

**DOI:** 10.1186/s13075-014-0443-y

**Published:** 2014-09-26

**Authors:** Megan Bohensky, Mark Tacey, Caroline Brand, Vijaya Sundararajan, Ian Wicks, Sharon Van Doornum

**Affiliations:** Melbourne EpiCentre, Department of Medicine, Royal Melbourne Hospital, Level 7 E Block, Parkville, VIC 3050 Australia; Melbourne EpiCentre, Department of Medicine, The University of Melbourne, Melbourne, Australia; Department of Medicine, St. Vincent’s Hospital, University of Melbourne, Melbourne, Australia; Department of Medicine, Southern Clinical School, Monash University, Melbourne, Australia; Rheumatology Unit, Melbourne Health & University of Melbourne, Melbourne, Australia; Inflammation Division, Walter & Eliza Hall Institute of Medical Research, Melbourne, Australia

## Abstract

**Introduction:**

To compare statin initiation and treatment non-adherence following a first acute myocardial infarction (MI) in patients with inflammatory rheumatic disease **(**IRD) and the general population.

**Methods:**

We conducted a retrospective cohort study using a population-based linked database. Cases of first MI from July 2001 to June 2009 were identified based on International Classification of Diseases (ICD-10-AM) codes. Statin initiation and adherence was identified based on pharmaceutical claims records. Logistic regression was used to assess the odds of statin initiation by IRD status. Non-adherence was assessed as the time to first treatment gap using a Cox proportional hazards model.

**Results:**

There were 18,518 individuals with an index MI over the time period surviving longer than 30 days, of whom 415 (2.2%) were IRD patients. The adjusted odds of receiving a statin by IRD status was significantly lower (OR =0.69, 95% CI: 0.55 to 0.86) compared to the general population. No association between IRD status and statin non-adherence was identified (hazard ratio (HR) =1.12, 95% CI: 0.82 to 1.52).

**Conclusions:**

Statin initiation was significantly lower for people with IRD conditions compared to the general population. Once initiated on statins, the proportion of IRD patients who adhered to treatment was similar to the general population. Given the burden of cardiovascular disease and excess mortality in IRD patients, encouraging the use of evidence-based therapies is critical for ensuring the best outcomes in this high risk group.

**Electronic supplementary material:**

The online version of this article (doi:10.1186/s13075-014-0443-y) contains supplementary material, which is available to authorized users.

## Introduction

People with inflammatory rheumatic diseases (IRD), including rheumatoid arthritis (RA), systemic lupus erythematosus (SLE), systemic sclerosis (SSc), spondyloarthritis (SpA), vasculitis and other chronic inflammatory diseases of the connective tissues, are known to have increased cardiovascular mortality and morbidity compared to the general population [1-9]. This phenomenon is thought to be related to chronic systemic inflammation that underlies the progression of typical atherosclerotic disease and may be accelerated in IRD sub-types [10]. Following a myocardial infarction (MI), RA patients have been shown to have increased fatality rates, with adjusted odds (ORs) ranging from 1.2 to 3.0 [11-13]. More recently, we have shown that IRD patients experiencing a first myocardial infarction (MI) have significantly increased adjusted odds of death at 30 days (OR 1.51, 95% CI 1.24, 1.84) when compared to the general population [14]. The majority of deaths were found to be due to cardiovascular causes.

Clinical trials have demonstrated the efficacy of secondary pharmacological prevention strategies for reducing cardiovascular morbidity and mortality in the general population [15-18]. Based on this evidence, current guidelines advocate the use of secondary prevention medications (anti-thrombotic therapy, oral beta-blockers, angiotensin-converting-enzyme (ACE) inhibitor and statins) following acute MI, where such therapy is not contra-indicated [19-21]. Despite this evidence, our previous research suggests that RA patients receive sub-optimal pharmacotherapy after an MI. In a detailed review of the medical records of 90 RA patients admitted with acute MI, we found treatment with *β*-blockers (OR 0.42, 95% CI 0.18, 0.96) and statins (OR 0.21, 95% CI 0.09, 0.46) to be significantly lower than matched controls without RA at discharge [22]. The reasons for differential treatment were not accounted for by contraindication to therapy in RA patients. As the study included only three hospitals (two tertiary public hospitals and one large metropolitan private hospital in Victoria, Australia) and was focused on in-hospital treatment only, it is unknown if these findings are generalisable to other settings. Subsequently Lindharsen *et al* conducted a population-based study of national registries in Denmark, which included 877 patients with RA after a first MI. They found RA patients had significantly lower odds of treatment with aspirin (OR 0.80, 95% CI:0.67, 0.96), *β*-blockers (OR 0.77, 95% CI 0.65, 0.92) and statins (OR 0.69, 95% CI 0.58, 0.82) [23].

As the therapeutic benefit of secondary pharmacological prevention therapies requires adherence, discontinuing treatment can have serious repercussions on patient outcomes. RA patients in one study had adherence rates of 45.4% to statin therapies over a 4-year period [24] with poor adherence increasing the risk of MI by 2% for each 1-month increase in the duration of non-adherence [25]. Poor adherence was also found to be associated with a significantly increased risk of cardiovascular mortality (hazard ratio (HR) 1.60, 95% CI 1.15, 2.23) and all-cause mortality (HR 1.79, 95% CI 1.46, 2.20) compared to RA patients who adhered to treatment [24].

Data on pharmacotherapy initiation and adherence are lacking for patients with IRD sub-types other than RA. Given the known burden of cardiovascular disease, mortality and increased fatality following an MI in IRD [14], the primary aim of the present study was to compare initiation and non-adherence to statin treatment following a first acute MI in patients with IRD, when compared to the general population.

## Methods

### Data sources

We used the Western Australian Data Linkage System (WADLS) to undertake this study. WADLS uses probabilistic data linkage methods to create and maintain a dynamic set of linkages across 30 administrative health datasets, including public and private hospital morbidity data, pharmaceutical claims records and death data [26]. As these data are administrative in nature, there are no missing data items. An evaluation of the WADLS linkage has shown that the probabilistic matching algorithm based on patient names and other partial identifiers has 99.9% sensitivity [26]. Information on patient diagnoses and procedures for each hospital episode are coded according to the International Statistical Classification of Diseases and Related Health Problems, 10th Revision, Australian Modification [27] (ICD-10-AM) beginning 1 July 1998 and ICD version 9, Canadian Modification (ICD-9-CM) prior to 1 July 1998. Pharmaceutical claims records were obtained from the Australian Pharmaceutical Benefits Scheme (PBS), which holds information on all subsidised prescription medicines provided in the outpatient setting [28].

### Definition of index MI and IRD

All cases of MI from 1 July 2001 to 30 June 2009 were identified based on the ICD-10-AM classifications (see Additional file 1 for a list of all codes used to undertake data analyses). An individual was considered to have experienced a first acute MI if they had a diagnosis of MI within this time period and no diagnosis of MI in the previous five years (that is, the dataset has records back to 1 July 1996 to provide a five-year look-back period). Our rationale for selecting a five-year look-back period has been described previously [29].

For the purposes of this study, IRD included the following diagnoses: RA, SLE, psoriatic arthritis (PsA), ankylosing spondylitis (AS), enteropathic arthritis (EA), systemic sclerosis (SSc), systemic vasculitis (SV), Sjogren’s syndrome (SjS), polymyalgia rheumatica (PMR), mixed connective tissue disease (MCTD), dermatomyositis (DM) and polymyositis (PM). Each of these conditions was considered to be present when the relevant ICD-9-CM or ICD-10-AM diagnosis codes (see Additional file 1) were recorded during the index MI admission or during any hospital admission in the 5 years prior to the index MI. We also examined the spondyloarthritis group, which comprised AS, PsA and EA. Some patients were found to have multiple recorded IRD conditions (n =80). These patients were counted only once in the primary analysis, but may have contributed data to more than one subgroup in the IRD subgroup analysis.

### Outcomes

Our outcomes were statin initiation and non-adherence. We chose to focus primarily on statins, as these are one of the most commonly used secondary prevention pharmacological therapies following MI in Australia [30].

As PBS records were provided in a month-year format, statin initiation was defined as having a statin dispensed (including atorvastatin, fluvastatin, pravastatin, rosuvastatin and simvastatin) within the month after the hospital separation for the MI. We excluded patients who died within 30 days of their hospital separation involving the MI (n =2,608, 12.3%), as we could not determine whether they had received a supply of statins in hospital. As prescribing behaviour may vary depending on whether a patient had received statins previously, we also conducted a stratified analysis of initiation to examine those patients who had a statin script prior to their MI (prior users) and those who had never been prescribed statins (statin-naïve patients).

For the assessment of non-adherence, we only considered those patients who were initiated on statins. Non-adherence was defined as having a gap between prescriptions exceeding three consecutive months during the follow-up period. This definition was based on the refill-sequence model of persistence, which has been described previously [31]. In our dataset, prescription dates were provided in a month-year format, therefore we permitted up to a 3-month gap in prescriptions to make allowances for people who may have filled a script at the end of one month, did not fill a script in the next month, but then did fill their next script at the beginning of the subsequent month (for example, completed a script in January and refilled in March).

### Statistical analysis

Descriptive statistics were used to describe the differences in the patient population for IRD patients compared with non-IRD patients. Potential predictors of statin initiation, non-adherence and mortality were considered in the descriptive analysis and time-to-event analysis. These covariates included age, gender, marital and indigenous status, socio-economic status, proximity to goods and services, use of statins prior to index MI, and comorbidities of interest (predominantly based on the Charlson comorbidity algorithm [32,33]). All covariates were binary. Age was initially considered as a continuous variable and then as a variable with five levels (that is, <50, 50 to 59, 60 to 69, 70 to 79 and ≥80 years).

The chi-squared test was used to identify significant differences between IRD and non-IRD patients for binary or categorical variables, the Fisher exact test was used where any cell values were under five, and the Mann-Whitney test was used to test for differences for continuous variables that were not normally distributed (that is, length of stay). Significance was set at *P* <0.05.

Statin initiation was assessed using a multivariate logistic regression analysis with adjustment for significant factors. Given the potential gain in efficiency of matching cases to controls, a sensitivity analysis was undertaken to explore this strategy. Non-IRD controls were matched to IRD cases on age and gender in a 5-to-1 ratio. Statin non-adherence was assessed as a competing-risk Cox proportional hazards model with death as the competing risk to statin non-adherence and time since index MI as the entry point into the model. Time-to-event analysis was censored at the last possible follow-up date of 30 June 2010.

Statistical analysis was performed using Stata version 12 (StataCorp LP, Texas, USA). The study was approved by the Melbourne Health Research Ethics Committee. As researchers accessed de-identified data only, patient consent was not considered necessary.

## Results

### Statin initiation

**Figure 1:**
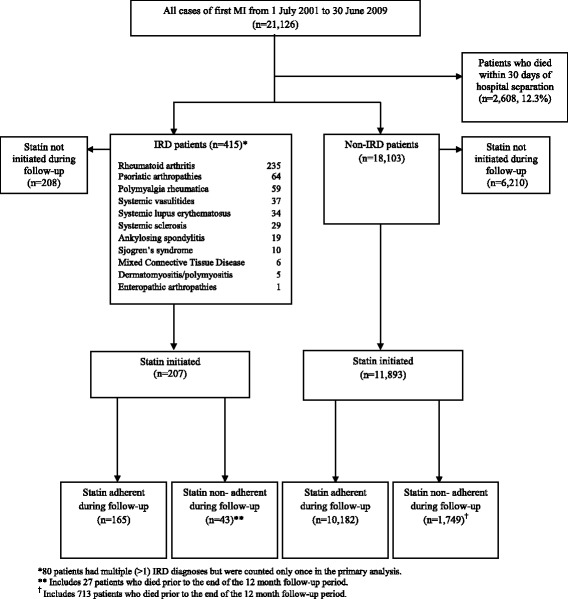
**Flowchart of patient inclusion for inflammatory rheumatic disease (IRD) and non-IRD patients with an index myocardial infarction (MI) from 1 July 2001 to 30 June 2009.**

There were 18,518 individuals with an index MI who survived longer than 30 days, of whom 415 (2.2%) were identified as IRD patients (Figure 1). Of these, there were 207 (49.9%) IRD patients initiated on statins compared to 11,893 (65.7%) non-IRD patients, which was a significantly different proportion (*P* <0.01) (Table 1). An additional file includes *P*-values for Table 1 (see Additional file 2).

**Table 1:** **Inflammatory rheumatic disease (IRD) and non-IRD patients by statin initiation**

**Variable**	**IRD patients (n =415)**				**Non-IRD patients (n =18,103)**			
	**Statins initiated**		**Statins not initiated**		**Statins initiated**		**Statins not initiated**	
	**n**	**%**	**n**	**%**	**n**	**%**	**n**	**%**
Number of patients	207	100	208	100	11,893	100	6,210	100
**Age, years**								
<50	9	4.4	12	5.8	1,445	12.2	613	9.9
50 to 59	32	15.5	18	8.7	2,368	19.9	804	12.9
60 to 69	38	18.4	21	10.1	2,748	23.1	947	15.3
70 to 79	77	37.2	60	28.9	3,085	25.9	1,367	22.0
80+	51	24.6	97	46.6	2,247	18.9	2,479	39.9
Female	112	54.1	149	71.6	3,853	32.4	2,784	44.8
Indigenous	1	0.5	7	3.4	338	2.8	376	6.1
Married/de-facto	117	56.5	93	44.7	7,888	66.3	3,188	51.3
High accessibility to services^1^	164	79.2	164	78.8	8,500	71.5	4,390	70.7
Accessibility/Remoteness Index of Australia (ARIA) - remote^2^	6	2.9	10	4.8	478	4.0	410	6.6
Lowest quartile of Socio-Economic Indexes for Areas Index of Relative Socio-Economic Disadvantage (SEIFA-IRSD)	11	5.3	16	7.7	805	6.8	547	8.8
Length of stay, median (IQR)	8	(5 to 16)	17	(7 to 42)	5	(4 to 10)	9	(5 to 24)
Statin use within 1 month prior to index MI	60	29.0	6	2.9	3,090	26.0	369	5.9
**Comorbidities at index admission** ^**3**^								
Cancer	12	5.7	8	3.9	340	2.9	550	8.9
Cerebral vascular accident	6	2.9	14	6.7	376	3.2	435	7.0
Dementia	3	1.4	11	5.3	108	0.9	183	2.9
Diabetes	35	16.9	45	21.7	1,803	15.2	1,194	19.2
Liver disease	0	0.0	3	1.5	18	0.2	63	1.0
Paraplegia	3	1.4	7	3.4	153	1.3	227	3.7
Peptic ulcer	3	1.4	10	4.8	110	0.9	110	1.8
Pulmonary disease	19	9.2	38	18.3	586	4.9	651	10.5
Peripheral vascular disease	3	1.4	6	2.9	274	2.3	202	3.3
Renal Disease	29	14.0	32	15.4	882	7.4	788	12.7
**Cardiovascular risk factors**								
Smoker	40	19.3	30	14.4	3,071	25.8	1,244	20.0
Hypertension	109	52.7	97	46.6	6,299	53.0	2,825	45.5
Hypercholesterolaemia	57	27.5	15	7.2	3,873	32.6	1,165	18.8
Arrhythmia	58	28.0	71	34.1	2,584	21.7	1,849	29.8
Obesity	16	7.7	7	3.4	865	7.3	313	5.0
**Procedure, during admission**								
PTCA	45	21.7	20	9.6	3,358	28.2	679	10.9
CABG	4	1.9	4	1.9	570	4.8	159	2.6
Count of medications at 1 months post MI, median (IQR)	7	(4 to 10)	3	(0 to 7)	4	(2 to 7)	0	(0 to 4)

Results are presented as number (n) and percent (%) unless stated otherwise. ^1^Based on ARIA code =1, major cities, highly accessible. ^2^Based on ARIA code = 4 (remote) and ARIA code = 5 (very remote). ^3^Based on Charlson comorbidity components and other risk factors. MI, myocardial infarction; CABG, coronary artery bypass graft; PTCA, percutaneous transluminal coronary angioplasty.

Within the IRD group, patients initiated on statins were significantly more likely to be aged under 70 years, male, have received statins in the month prior to MI, have hypercholesterolaemia or be obese, but were less likely to be indigenous or have certain comorbidities, including dementia, peptic ulcer disease and pulmonary disease when compared to patients not initiated on statins. IRD patients initiated on statins also received a significantly higher median number of medications at 1 month post MI than patients not initiated on statins.

After adjustment for age, gender, socio-economic status, geographic location and relevant co-morbidities, the odds of IRD patients receiving a statin were significantly reduced at 0.67 (95% CI 0.54, 0.84). As cardiovascular risk factors are known to be under-reported in administrative data [34], we conducted a sensitivity analysis where all cardiovascular risk factor variables (hypercholesterolaemia, arrhythmia, obesity, hypertension and smoking) were removed from the statin initiation multivariate analysis. We identified very little change in the primary outcome (OR 0.65, 95% CI 0.52 to 0.81, *P*-value <0.001 from the estimated OR 0.67, 95% CI 0.54 to 0.84, *P*-value =0.001). We retained cardiovascular risk factors in the final model as the standard error for the IRD variable in the model without risk factors was increased. In our additional sensitivity analysis, where IRD patients were matched on a 1:5 ratio with non-IRD patients, the OR for IRD patients receiving a statin was 0.72 (95% CI 0.56, 0.92, *P*-value =0.008) indicating little change in the log odds of statin initiation.

Statin initiation by IRD sub-type is reported in Table 2. Individuals with SpA (OR 0.43, 95% CI 0.26, 0.70), SV (OR 0.44, 95% CI 0.20, 0.94) and SLE (OR 0.24, 95% CI 0.11, 0.59) had significantly lower adjusted odds of being initiated on a statin compared to people without IRD. Apart from SjS, the adjusted ORs for all other IRD sub-types were <1.0 but were not statistically significant. Given that there can be a delay in the uptake of guidelines, we also examined statin initiation over two time periods. Statin initiation increased in the period from 1 July 2004 to 30 June 2009 compared to the period from 1 July 2002 to 30 June 2004. After adjusting for significant factors, the OR for statin initiation among IRD patients in the first period (from 1 July 2002 to 30 June 2004) was 0.64, 95% CI 0.45, 0.91) and in the latter period (1 July 2004 to 30 June 2009) it was 0.72, 95% CI 0.53, 0.98).

**Table 2:** **Crude and adjusted odds ratios (ORs) for statin initiation by inflammatory rheumatic disease (IRD) sub-type**

**Condition**	**Count**	**Crude OR**	**Adjusted OR**
Rheumatoid arthritis	235	0.58 (0.45 to 0.75)	0.78 (0.58 to 1.05)
Polymyalgia rheumatica	59	0.38 (0.23 to 0.64)	0.73 (0.41 to 1.29)
**Spondyloarthritis**	**84**	**0.41 (0.27 to 0.63)**	**0.43 (0.26 to 0.70)**
Ankylosing spondylitis	19	0.72 (0.29 to 1.79)	0.54 (0.19 to 1.52)
**Psoriatic arthropathies**	**64**	**0.36 (0.22 to 0.59)**	**0.42 (0.24 to 0.73)**
Enteropathic arthropathies	1	-	-
Systemic necrotising vascultis	37	0.32 (0.16 to 0.62)	**0.44 (0.20 to 0.94)**
**Systemic lupus erythematosus**	**34**	**0.22 (0.10 to 0.46)**	**0.24 (0.11 to 0.59)**
Systemic sclerosis	29	0.56 (0.27 to 1.16)	0.44 (0.20 to 1.01)
Sjogren’s syndrome	10	2.09 (0.44 to 9.84)	4.08 (0.64 to 26.04)
Dermatomyositis and polymyositis	5	0.78 (0.13 to 4.69)	0.86 (0.11 to 7.04)
Mixed Connective Tissue Disease	6	0.26 (0.05 to 1.43)	0.20 (0.03 to 1.52)
**Total inflammatory rheumatic diseases**	**415**	**0.52 (0.43 to 0.63)**	**0.67 (0.54 to 0.84)**

Adjusted ORs were adjusted for age, gender, socio-economic status, geographic location and relevant co-morbidities. For sub-type, 80 patients had multiple (>1) IRD diagnoses but were counted only once in the primary analysis. Bolded text indicates statistically significant factors (*P* <0.05).

There were 14,360 statin-naïve patients and 3,450 patients with at least one statin prescription prior to their MI. In our stratified analysis, the odds of statin initiation in statin naïve IRD patients was significantly reduced (OR =0.64, 95% CI 0.50 to 0.81). For those who used statins priors to their MI, the estimated odds for continued statin use was not significantly different (*P* =0.578) in the IRD group compared to the non-IRD group (OR 1.28, 95% CI 0.53, 3.07).

### Statin non-adherence

The rate of statin non-adherence within 12 months post-MI for patients with IRD was 20.7% compared to 14.7% for patients without IRD (*P* =0.02). However, this includes patients who died over the follow-up period and the mortality rate was higher for the IRD group (13.0% compared to 6.0% for the non-IRD group, *P* <0.01). After adjusting for age, gender, marital status, indigenous status, socio-economic status, proximity to goods and services, prior use of statins, relevant comorbidities and accounting for death as a competing risk, no association between IRD and statin non-adherence was identified (HR 1.11, 95% CI 0.82, 1.52, Table 3). No significant associations were identified between statin non-adherence and IRD sub-type.

**Table 3:** **Factors associated with the rate of statin non-adherence (n =12,100)**

	**Hazard ratio**	**95% CI**	***P*** **-value**
Inflammatory rheumatic disease	1.11	0.82, 1.52	0.49
**Age group, years**			
<50	1	-	-
**50 to 59**	**0.80**	**0.72, 0.89**	**<0.001**
**60 to 69**	**0.67**	**0.59, 0.75**	**<0.001**
**70 to 79**	**0.58**	**0.51, 0.66**	**<0.001**
**80+**	**0.49**	**0.42, 0.58**	**<0.001**
Female	0.97	0.89, 1.06	0.49
**Indigenous**	**2.01**	**1.69, 2.41**	**<0.001**
**Married/de-facto**	**0.91**	**0.84, 0.99**	**0.03**
Remote^1^	1.02	0.86, 1.22	0.86
Lowest quartile of Socio-Economic Indexes for Areas Index of Relative Socio-Economic Disadvantage (SEIFA-IRSD)	1.12	0.97, 1.30	0.12
Elective admission	0.87	0.75, 1.01	0.06
Intensive Care Unit during admission	0.98	0.87, 1.09	0.65
Hospital type			
Tertiary	1	-	-
Public metro	0.92	0.65, 1.31	0.64
**Rural public/private**	**0.88**	**0.77, 0.99**	**0.04**
Private metro	1.00	0.89, 1.13	0.94
Comorbidities at index admission^2^			
Cancer	1.09	0.77, 1.53	0.63
Cerebral vascular accident	0.80	0.58, 1.09	0.16
Dementia	1.04	0.60, 1.81	0.88
Diabetes	1.02	0.91, 1.14	0.72
HIV	-	-	-
Liver disease	1.51	0.63, 3.63	0.36
Paraplegia	0.92	0.56, 1.53	0.76
Peptic ulcer	1.29	0.86, 1.94	0.21
Pulmonary disease	1.06	0.87, 1.29	0.57
Peripheral vascular disease	1.02	0.78, 1.35	0.87
Renal Disease	1.00	0.84, 1.19	0.96
Cardiovascular Risk factors			
**Smoker**	**1.17**	**1.07, 1.27**	**<0.001**
Hypertension	1.06	0.98, 1.14	0.15
Hypercholesterolaemia	1.03	0.95, 1.11	0.54
**Obesity**	**1.15**	**1.01, 1.30**	**0.03**
Count of PBS medications at 3 months post MI			
0 to 1	1	-	-
**2 to 5**	**0.76**	**0.60, 0.96**	**0.02**
**6 to 10**	**0.70**	**0.55, 0.89**	**<0.01**
**>10**	**0.68**	**0.52, 0.87**	**<0.01**
**Statin use within 12 months prior to index MI**	**0.90**	**0.82, 0.98**	**0.02**

Analysis of factors was based on a competing risk, Cox Regression Model, adjusting for death for all patients initiated on statins. ^1^Based on ARIA Code =4, excluding those deceased within 3 months, unlimited follow-up. ^2^Based on Charlson comorbidity components. Bolded text indicates statistically significant factors (*P* <0.05). PBS, Pharmaceutical Benefits Scheme; MI, myocardial infarction.

## Discussion

In a cohort of 21,126 people experiencing a first MI over an eight-year period, including 382 people with IRD, we found approximately 50% of IRD patients were not initiated on statins within the first month following MI. This translates to an adjusted OR of 0.67. Among patients who were initiated on statins, the proportion of patients who adhered to treatment was very good (80%) and not significantly different from the general population, after accounting for the higher mortality in IRD patients. Given the high burden of cardiovascular disease and mortality in IRD patients, ensuring the prompt initiation and continuation of evidence-based therapies is critical for ensuring the best outcomes.

One explanation for the lower rates of statin initiation in IRD patients may be the non-traditional presentation of cardiovascular (CV) risk in this group. IRD patients who were already receiving statins prior to their AMI did not have significantly lower rates of continuation when compared to non-IRD patients. This suggests that the issue with statin initiation relates to identifying and treating statin-naïve IRD patients. Previous guidelines have recommended statin treatment be initiated after MI only when total cholesterol is greater than 5 mmol/l [35]. Changes to guidelines and practice started to occur in the early 2000s when statin use was recommended regardless of lipid levels [36], but it may have taken several years for this recommendation to gain widespread adoption by clinicians.

Patients with SLE, SV and SpA were found to receive less statin treatment compared to the other IRD groups. These patients are known to have a two- to three-fold risk of CV mortality compared to the general population and this risk may be further increased in the presence of traditional CV risk factors [37-39]. Statin therapy is recommended due to its lipid-lowering properties and anti-inflammatory effects [37,40]. However, several reports in the literature have suggested that long-term statin exposure may trigger or aggravate autoimmune diseases, particularly SLE, due to immunomodulatory effects [41]. While the evidence for a causal relationship between statin use and SLE is not well-established, it is unclear if clinicians are hesitant to initiate statin therapy in SLE patients due to this concern. There was also a higher proportion of indigenous Australians in the SLE group (17.7%) [42], and disparities in statin treatment for indigenous populations have been noted previously [43]. A recent study has shown that classic CV risk scores underestimate the risk for patients with PsA when compared with ultrasound assessment of carotid intima thickness, which may contribute to under-treatment in this population [44]. Given the heightened risk of CV disease morbidity and mortality in patients with SLE, SV and SpA, further research is required to understand barriers to statin initiation in these populations.

In their study of initiation and adherence to secondary prevention pharmacotherapies, Linhdardsen *et al*. found RA patients in Denmark were less likely to be initiated on and adhere to statin therapies following MI [23]. These authors also suggest the added clinical complexity of RA may discourage clinicians from initiating statin treatment, which carries risks of myopathy, rhabdomyolysis, and hepatotoxicity. We also identified lower rates of statin adherence among our IRD cohort, but this was not statistically significant, which may relate to our smaller sample size or to social differences between Denmark and Australia. While prescription of lipid-lowering agents was 79% less (OR 0.21, 95% CI 0.09, 0.46) in our previous study of RA patients over the period from 1995 to 2005 [22], in the present study we found prescription of statins to be 33% lower in IRD patients over the period from 2001 to 2009. Although our data were taken from a different state in Australia, this observation could suggest changing practice over time, as we identified an increasing trend in statin initiation for IRD patients by time period. This trend may coincide with guidelines on the management of acute coronary syndrome published by the Australian National Heart Foundation in 2006, which recommend the use of medications at discharge, including statins, in long-term management after control of the MI [20].

This study included a large, population-based cohort including all incident MIs within the state of Western Australia, however there are several limitations worth noting. It is not known if patients received statins during their hospital admission or had a supply dispensed at the time of discharge, as PBS records do not record hospital drug dispensing. We also assumed that if prescriptions were filled, then the drugs were taken, but this may not always be so. The data on prescription dates were in a month-year format, rather than precise dates. Therefore, we had to make assumptions about the exact duration of scripts and period of adherence. We accepted three months as being an acceptable gap between scripts; however, accepting a full 90-day gap between prescriptions may underestimate non-adherence. The study relied on coded hospital administrative data, which were not designed primarily for research purposes and may therefore contain coding errors. While CV risk factors are known to be under-coded in administrative data, removal of these factors from the multivariate analysis did not change our findings. We chose a five-year look-back period to identify a history of previous MIs. While this may have misclassified some subsequent MIs as the first MI, an individual who experienced an MI during the study period and had a history of previous MI more than five years prior would be likely to receive comparable treatment to individuals with a true first MI [45,46]. Furthermore, this misclassification is likely to be equal in the IRD and non-IRD groups and therefore not introduce bias. Similarly, the coding of IRD conditions may have inaccuracies and be biased towards only coding the most severe cases. However, we chose a three-year look-back period to capture IRD conditions that may have been coded in a patient’s previous hospital admissions. As our primary analyses examined initiation and non-adherence in IRD patients overall, this may lead to an over-generalisation of findings from one rheumatic disease group to all groups. While we also considered initiation and non-adherence among the IRD sub-types, there was a limited sample size in some populations. The presence of multiple IRD sub-types may be due to coding errors over time or diagnostic uncertainty. As data were de-identified, we were unable to validate diagnoses against a secondary source of information and exclude diagnoses due to potential misclassification.

## Conclusion

Despite recent improvements in statin initiation rates, this large population-based study found that patients with IRD were less likely to be initiated on statin therapies following an MI when compared to the general population. However, once initiated, IRD patients were found to adhere to statins at similar rates to non-IRD patients. Given the high risk of CV disease and mortality in IRD populations, increased rates of early initiation on statins following MI as an effective and safe secondary preventative therapy should be encouraged. The underlying factors driving this differential treatment should be examined further to improve care and outcomes of patients with IRD.

## Additional files

Additional file 1:
**ICD-9-CM and ICD-10-AM classification codes.** This document provides the International Classification of diseases codes that were used to identify disease groups, procedures and comorbidities. Additional file 2: Table S1. This document provides the data in a table with *P*-values to demonstrate the statistical differences between patients initiated on statins or not.

## Abbreviations

ACE: angiotensin-converting-enzyme
AS: ankylosing spondylitis
CV: cardiovascular
DM: dermatomyositis
EA: enteropathic arthritis
HR: hazard ratio
ICD-10-AM: International Classification of Diseases version 10: Australian Modification
ICD-9-CM: International Classification of Diseases version 9: Canadian Modification
IRD: inflammatory rheumatic disease
MCTD: mixed connective tissue disease
MI: myocardial infarction
OR: odds ratio
PBS: Pharmaceutical Benefits Scheme
PM: polymyositis
PMR: polymyalgia rheumatica
PsA: psoriatic arthritis
RA: rheumatoid arthritis
SjS: Sjogren’s syndrome
SLE: systemic lupus erythematosus
SpA: spondyloarthritis
SSc: systemic sclerosis
SV: systemic vasculitis
WADLS: Western Australian Data Linkage System
