# Supplementary material for: Statin initiation and treatment non-adherence following a first acute myocardial infarction in patients with inflammatory rheumatic disease versus the general population
Source: Arthritis Res Ther. 2014 Sep 26;16(5):443. doi: 10.1186/s13075-014-0443-y (PMC4201728; doi:10.1186/s13075-014-0443-y)
Supplement: Additional file 1: — ICD-9-CM and ICD-10-AM classification codes. This document provides the International Classification of diseases codes that were used to identify disease groups, procedures and comorbidities. [file 13075_2014_443_MOESM1_ESM.docx]

**Additional file 1: ICD-9-CM and ICD-10-AM classification codes (web only)**

| **Condition Type** | **ICD-9-CM Codes (admissions prior to 1 July 1998)** | **ICD-10-AM Codes (admissions from 1 July 1998)** |
| --- | --- | --- |
| **Diagnoses** | | |
| **Myocardial Infarct (MI)** |  |  |
| Myocardial Infarct Causes of Death |  | I20.xx, I21.xx, I22.xx, I23.xx, I24.xx, I25.xx, I26.xx, I3.xx, I4x.xx, I5.xx, I6x.xx, I7x.xx, I81.xx and I82.xx |
| **IRD Conditions** |  |  |
| Seropositive and Other Rheumatoid Arthritis (RA) | 7140x, 7141x, 7142x, 7143x, 71481, 71489, 7149x | M05.xx, M06.0x, M06.2x, M06.3x, M06.8x, M06.9x |
| Systemic lupus erythematosus (SLE) | 7100x | M32.xx |
| Systemic sclerosis (SSc) | 7101x | M34.xx |
| Psoriatic arthropathies (PsA) | 6960x, 7140x, 71430 | L40.5x, M07.0x, M07.1x, M07.2x, M07.3x, M09.0x |
| Ankylosing Spondylitis (AS) | 7200x | M08.1x, M45.xx |
| Enteropathic arthropathies (EA) | 7131x, 71430 | M07.4x, M07.5x, M07.6x, M09.1x, M09.2x |
| Systemic necrotizing vasculitides (SNV) | 4460x, 44620, 44621, 44629, 4464x, 4465x, 4466x, 4467x | M30.0x, M30.1x, M30.2x, M30.8x, M31.0x, M31.1x, M31.3x, M31.4x, M31.5x, M31.6x, M31.7x, M31.9x |
| Dermatolymyositis (DMPM) | 7103x, 7104x | M33.0x, M33.1x, M33.2x, M33.9x, M36.0x |
| Sjogren syndrome (SjS) | 7102x | M35.0x |
| Systemic sclerosis (SSc) | 7101x | M34.xx |
| Mixed Connective Tissue Disease (MCTD) | 2794x, 7108x, 7109x | M35.1x, M35.8x, M35.9x |
| Polymyalgia rheumatica (PMR) | 4465x, 725xx | M31.5x, M35.3x |
| **Procedures** | | |
| Percutaneous transluminal coronary angioplasty |  | 35303-06, 35303-07, 35304-00, 35305-00, 35309-06, 35309-07, 35309-08, 35309-09, 35310-00, 35310-01, 35310-03, 35310-04 and 35315-00 |
| Coronary Artery Bypass Graft (CABG) |  | 38497-00, 38497-01, 38497-02, 38497-03, 38497-04, 38497-05, 38500-00, 38500-01, 38500-02, 38500-03, 38500-04, 38503-00, 38503-01, 38503-02, 38503-03, 38503-04 and 38637-00 |
| **Charlson & additional comorbidities** | | |
| Cancer |  | C0x.xx, C1x.xx, C2x.xx, C3x.xx, C40.xx, C41.xx, C43.xx, C45.xx, C46.xx, C47.xx, C48.xx, C49.xx, C5x.xx, C6x.xx, C71.xx, C72.xx, C73.xx, C74.xx, C75.xx, C76.xx, C80.xx, C81.xx, C82.xx, C83.xx, C84.xx, C85.xx, C88.3x, C88.7x, C88.9x, C90.0x, C90.1x, C91.xx, C92.xx, C93.xx, C94.0x, C94.1x, C94.2x, C94.3x, C94.51, C94.7x, C95.xx, C96.xx |
| Congestive Heart Failure |  | I50.xx |
| Connective Tissue Disorder |  | Not used as AIRD categories defined above and used as key exposure variable |
| Cerebral Vascular Accident |  | G45.0x, G45.1x, G45.2x, G45.4x, G45.8x, G45.9x, G46.xx, I60.xx, I61.xx, I62.xx, I63.xx, I64.xx, I65.xx, I66.xx, I67.0x, I67.1x, I67.2x, I67.4x, I67.5x, I67.6x, I67.7x, I67.8x, I67.9x, I68.1x, I68.2x, I68.8x, I69.xx |
| Dementia |  | F00.xx, F01.xx, F02.xx, F05.1x |
| Dementia with complications |  | E10.2x, E10.3x, E10.4x, E11.2x, E11.3x, E11.4x, E13.2x, E13.3x, E13.4x, E14.2x, E14.3x, E144.4x |
| Diabetes |  | E10.1x, E10.5x, E10.9x, E11.1x, E11.5x, E11.9x, E13.1x, E13.5x, E13.9x, E14.1x, E14.9x |
| HIV |  | B20.xx, B21.xx, B22.xx, B23.xx, B24.xx |
| Liver Disease |  | K70.2x, K70.3x, K71.7x, K73.xx, K74.0x, K74.2x, K74.3x, K74.4x, K74.5x, K74.6x |
| Metastatic Cancer |  | C77.xx, C78.xx, C79.xx, C80.xx |
| Acute myocardial infarction |  | Not used as key inclusion criteria for this study. |
| Paraplegia |  | G04.1x, G81.xx, G82.0x, G82.1x, G82.2x |
| Peptic ulcer |  | K25.xx, K26.xx, K27.xx, K28.xx |
| Pulmonary disease |  | J40.xx, J41.xx, J42.xx, J43.xx, J44.xx, J45.xx, J46.xx, J47.xx, J60.xx, J61.xx, J62.xx, J63.xx, J64.xx, J65.xx, J66.xx, J67.xx |
| Peripheral vascular disease |  | I71.xx, I73.9x, I79.0x, IR02.xx, Z95.8x, Z95.9x |
| Renal disease |  | N01.xx, N03.xx, N05.2x, N05.3x, N05.4x, N05.5x, N05.6x, N07.2x, N07.3x, N07.4x, N18.xx, N19.xx |
| Severe Liver Disease |  | K72.1x, K72.9x, K76.6x, K76.7x |
| Smoker |  | F17.1x, F17.2x, F17.3x, F17.4x, V15.82, V15.83, Z72.0x |
| Hypertension (uncomplicated) |  | I10.xx |
| Hypercholesterolaemia |  | E78.0x, E78.5x |
| Arrythmia |  | I44.xx, I45.xx, I47.xx, I48.xx, I49.xx |
